# Supplementary material for: Deterministic and Probabilistic Dietary Exposure Assessment to Deoxynivalenol in Spain and the Catalonia Region
Source: Toxins (Basel). 2022 Jul 20;14(7):506. doi: 10.3390/toxins14070506 (PMC9316368; doi:10.3390/toxins14070506)
Supplement: Supplementary file 1 [file toxins-14-00506-s001.zip › toxins-1819913-supplementary.pdf]

Supplemental Materials

Table S1. Normalised consumption (g/kg bw/day) of cereal-based products for the population under 18 years old from ENALIA survey for all of Spain. Mean levels and standard deviation (SD) were computed considering only consumers.

Food	Age group	N total	% cons	Mean	SD
White bread	0-11 months	182	43%	1.13	0.82
	12-35 months	333	79%	1.81	1.21
	3-9 years old	589	95%	1.59	0.88
	10-17 years old	627	97%	1.15	0.61
Whole bread	0-11 months	182	1%	0.89	0.41
	12-35 months	333	2%	3.64	3.14
	3-9 years old	589	6%	1.44	0.77
	10-17 years old	627	9%	0.98	0.60
Bread rusks	0-11 months	-	-	-	-
	12-35 months	333	4%	0.78	0.74
	3-9 years old	589	5%	0.56	0.47
	10-17 years old	627	3%	0.33	0.15
Crackers	0-11 months	182	5%	0.31	0.11
	12-35 months	333	12%	0.98	2.20
	3-9 years old	589	5%	0.92	0.96
	10-17 years old	627	5%	0.55	0.64
Cookies	0-11 months	182	81%	1.04	0.55
	12-35 months	333	74%	1.37	0.83
	3-9 years old	589	48%	1.27	0.78
	10-17 years old	627	34%	0.79	0.50
Other cookies	0-11 months	-	-	-	-
	12-35 months	333	1%	1.54	0.95
	3-9 years old	589	3%	1.62	1.10
	10-17 years old	627	2%	1.30	1.12
Muffins	0-11 months	182	1%	3.33	-
	12-35 months	333	10%	2.55	1.06
	3-9 years old	589	16%	1.73	0.79
	10-17 years old	627	16%	1.08	0.59
Cake	0-11 months	-	-	-	-
	12-35 months	333	0.30%	7.89	-
	3-9 years old	589	1%	3.91	1.93
	10-17 years old	627	2%	2.06	0.83
Breakfast cereals	0-11 months	182	2%	2.92	3.18
	12-35 months	333	3%	1.75	1.35
	3-9 years old	589	9%	1.13	0.95
	10-17 years old	627	8%	0.69	0.49
Pasta	0-11 months	182	9%	2.28	1.60
	12-35 months	333	49%	2.38	1.70
	3-9 years old	589	58%	1.76	1.32
	10-17 years old	627	59%	1.10	0.92

Table S2. Recovery percentages and limit of detection (LOD) of the method used for the determination of the analysed mycotoxins.

Mycotoxin	ppb	Recovery (%)	LOD (µg/kg)
DON	200	108.9	2
	300	106.8	2
	400	108.3	2
	600	107.6	2
	800	104.6	2
15-Ac-DON	12.5	104.0	3
	18.75	105.1	3
	25	113.3	3
	37.5	106.7	3
	50	101.9	3
3-Ac-DON	25	102.6	3
	37.5	100.6	3
	50	102.0	3
	75	107.9	3
	100	105.0	3
3GDON	20	102.6	10
	30	100.3	10
	40	102.5	10
	60	101.1	10
	80	100.2	10

Abbreviations: 3-Ac-DON, 3-acetyl-deoxynivalenol; 15-Ac-DON, 15-acetyl-deoxynivalenol; DON, Deoxynivalenol; 3GDON, Deoxynivalenol-3-glucoside; LOD, Limit of Detection

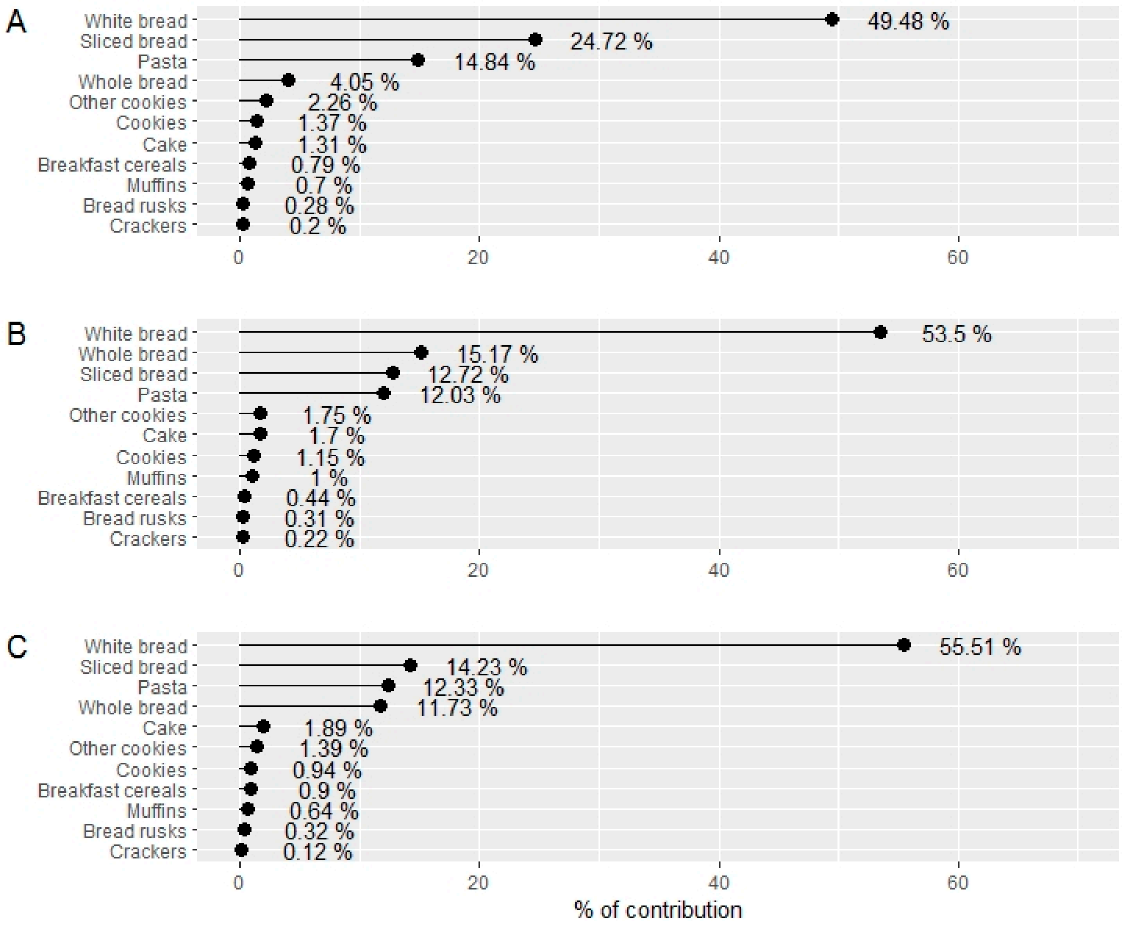

Figure S1. Contribution of cereal-based products to the global exposure to deoxynivalenol among adults from Spain. The exposure was estimated with the deterministic method for the 18–40 years old group (**A**), 41–60 years old group (**B**) and > 60 years old group (**C**).

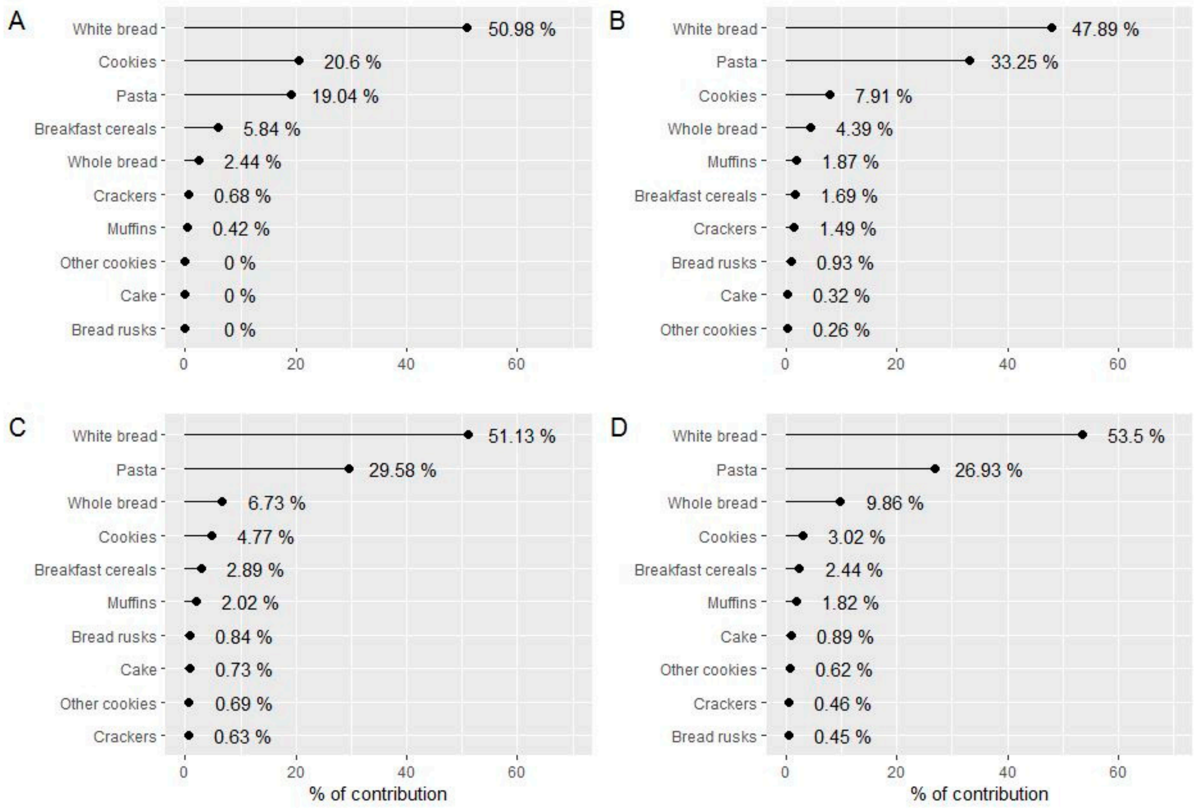

Figure S2. Contribution of cereal-based products to the global exposure to deoxynivalenol among children and adolescents from Spain. The exposure was estimated with the deterministic method for the 0–11 months old group (**A**), 12–35 months old group (**B**), 3–9 years old group (**C**) and for 10–17 years old group (**D**).
